# Analysis of methanol and ethanol in virgin olive oil

**DOI:** 10.1016/j.mex.2014.09.002

**Published:** 2014-09-23

**Authors:** Raquel B. Gómez-Coca, Rosario Cruz-Hidalgo, Gabriel D. Fernandes, María del Carmen Pérez-Camino, Wenceslao Moreda

**Affiliations:** a Instituto de la Grasa – CSIC, Avda. Padre García Tejero 4, E-41012 Sevilla, Spain; b Fats and Oils Laboratory, Faculty of Food Engineering, University of Campinas, 13083-970 Campinas, SP, Brazil

**Keywords:** Analysis of static headspace virgin olive oil volatiles, Virgin olive oil volatiles, Methanol determination, Ethanol determination, Fatty acid alkyl esters (FAAE), Olive oil, Analysis of volatiles

## Abstract

This work provides a short and easy protocol that allows the analysis of both methanol and ethanol in the static headspace of olive oil. The procedure avoids any kind of sample pre-treatment beyond that of heating the oil to allow a maximum volatile concentration in the headspace of the vials. The method's LOD is 0.55 mg kg^−1^ and its LOQ is 0.59 mg kg^−1^. Advantages of this method are:

• Simultaneous determination of methanol and ethanol (the pre-existing Spanish specification UNE-EN 14110 only analyses methanol).
• No need of equipment modifications (standard split injectors work perfectly). Use of a highly polar capillary GC column, leading in most cases to chromatograms in which only three dominant peaks are present – methanol, ethanol, and propanol (that is extremely positive for easy interpretation of results).
• Use of an internal standard (1-propanol) to determine the concentration of the analytes, reducing the presence of error sources.

## Graphical abstract

**Figure:**
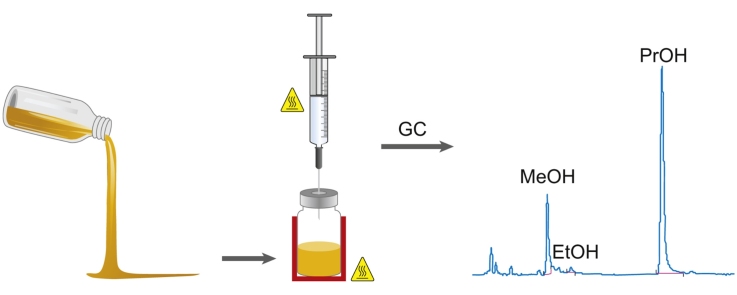

## Method details

The presence of short chain alcohols in virgin olive oil could be closely related with oil quality. Actually low amounts of methanol (MeOH) and ethanol (EtOH) are accepted since small quantities of these alcohols may be formed during the maturation of olives. On the other hand, high volumes of EtOH appear during the fermentation processes occurred mainly throughout olive fruit storage. The role of these short-chain alcohols regarding olive oil quality is still unclear, although their influence on the presence of fatty acid alkyl esters (FAAE), a quality parameter, is well known [1,2].

Due to the high volatility of short-chain alcohols their determination is normally accomplished by static headspace extraction followed by gas chromatography (GC) analysis [3,4].

### Reagents and samples

EtOH, MeOH, and 1-propanol (PrOH) used as reference materials were supplied by Romil Ltd. (Waterbeach, Cambridge, GB) and were of analytical quality.

Varietal virgin olive oils of Adramitini, Blanqueta, Bouteillan, Chemdal Kabilye, Cipresino, Coratina, Frantoio, Koroneiki, Leccino, Lechín de Granada, Manzanilla, Negral, Pendolino, Picual, Rapasayo, and Sigoise were directly prepared in the laboratory using the Abencor^®^ system described elsewhere [5] to assure maximum oil quality. Olive fruits were obtained from an irrigated orchard (drip irrigation) in the southern part of Spain, under optimal cultivation parameters. They were handpicked and, according to their maturity index, belonged to the categories 0–4 (deep green to black skin olives with white flesh) [6].

Chemically refined olive-pomace oil was obtained directly from the producer. This oil, together with all reagents and samples was kept in the dark at 4 °C until use.

Concentrated solutions of PrOH (internal standard, IS) were prepared by dissolving PrOH (cooled down to 4 °C, density = 0.810 g mL^−1^) in refined olive-pomace oil at proportions of 12.5 mL PrOH per kilo oil. From these concentrated solutions diluted IS solutions were prepared by mixing 1 g concentrated solution with 24 g refined olive-pomace oil. All critical volumes were measured with calibrated precision pipettes (0.6 μL systematic error). Both concentrated and diluted IS solutions were kept in the dark at −20 °C before use.

Samples were prepared just before the analysis in the following way: 3.00 g oil (room temperature) and 300 mg diluted IS solutions (room temperature) were introduced into a 9 mL vial (20 mm × 46 mm), which was immediately sealed with an aluminium crimp cap with silicone septa and with a PTFE face to eliminate bleed from the rubber portion. They were heated in a dry heat bath at 110 °C during 60 min. The vial headspace was then sampled via a thermostated stainless steel syringe (110 °C; sampling time = 30 s) and analysed by injecting the sample into the gas chromatograph.

After each injection the syringe was cleaned by blowing out air and then dry nitrogen. Blank injections were carried out after each analysis to check the absence of carry-over effects.

### Instrumentation

Heating of the samples was carried out in a Tembloc thermostat dry-block (JP Selecta S.A., Barcelona, Spain).

GC analyses of the volatiles were done with an Agilent 7890B Gas Chromatograph (Agilent Technologies, Santa Clara, California) equipped with a Tracer MHS123 2t^®^ Head Space Sampler and a flame ionization detector (FID). Acquisition of data was done with the Agilent ChemStation for GC System program. The conditions for the GC assays were: SP2380 column (poly 90% biscyanopropyl–10% cyanopropylphenyl siloxane), 60 m length × 0.25 mm internal diameter × 0.20 μm film (Sigma–Aldrich Co. LLC, St. Louis, MO, USA), 500 μL injection volume, hydrogen carrier gas at 1.5 mL min^−1^ and split injection (50:1 split ratio). The oven temperature programme was: 50 °C (7 min initial time), then rise at 10 °C min^−1^ to 150 °C and hold 3 min. The injector and detector temperatures were 150 °C and 170 °C, respectively.

### Development of the method

Tests to develop the method were performed using an in-house blank oil labelled as *refined olive-pomace oil* (oil comprising exclusively olive-pomace oils that have undergone classical refining), which had showed no significant chromatographic peaks within the retention time (Rt) windows of any of the volatiles under study. This oil was spiked with MeOH, EtOH, and PrOH (IS) at concentrations between 4 and 12 mg kg^−1^. On each case we observed three distinctive sharp, symmetrical peaks with a signal-to-noise ratio of at least 3 and with no tailing or shoulders corresponding to those three volatiles.

The peaks were identified by their absolute and relative Rt, which were the result of 34 injections. Always the absolute Rt was measured to three decimal places. These results also allowed the establishment of the Rt window for each target analyte to compensate the shifts in absolute Rt as a result of chromatographic variability. The relative Rt values kept constant all over the study. Those values and the Rt windows for both MeOH and EtOH, together with that for the IS are shown in Table 1.

Trials to establish the limit of detection (LOD), the limit of quantitation (LOQ), and differences in the response of the three volatiles were carried out by spiking eleven samples of refined olive-pomace oil with MeOH, EtOH and PrOH standard solutions at increasingly lower concentrations (from 992.1 mg kg^−1^ to 0.02 mg kg^−1^). The accepted concentration values were those that produced sharp, symmetrical analyte peaks with a signal-to-noise ratio of at least 2 and with no tailing or shoulders. Measures were always made in duplicate.

Since the lowest sensitivity –minimum concentration of analyte that could be measured and reported with an acceptable confidence that it was higher than zero– was that of PrOH, we decided to be conservative and accept the same limits for both MeOH and EtOH. In this way, hundred per cent of the spiked samples gave signals within the acceptance criteria and clearly distinguishable from the background. We set the LOD at 0.55 mg kg^−1^ for the species tested.

The empirical LOQ is defined as the lowest concentration at which the acceptance criteria are met and the quantitative value is within ±20% of the target concentration [7]. According to our results on virgin olive oil the EtOH lowest concentration to be expected is around 0.64 mg kg^−1^ (±0.12), however the fact of being unique and having such a ‘high’ RSDr (19%) made us take our second lowest value (0.74 mg kg^−1^) as reference for the calculation of the LOQ. Applying the aforementioned reasoning we set the LOQ for any of the volatiles under study at 0.59 mg kg^−1^.

### Analysis of samples

**Fig. 1:**
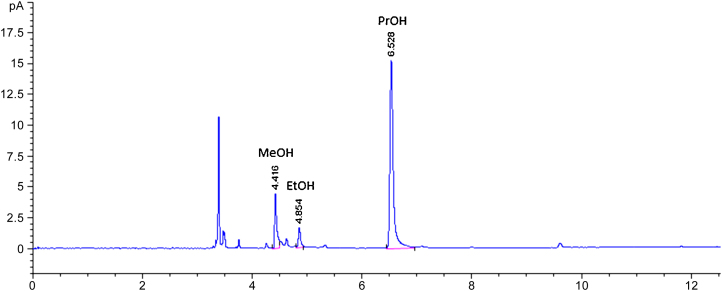
GC-FID chromatogram for methanol (MeOH) and ethanol (EtOH), together with 1-propanol (PrOH) used as internal standard, in virgin olive oil. This chromatogram has been obtained after analysing the sample according to the proposed method.

The volatile composition of 16 virgin olive oil varieties was determined according to the described method. The GC-FID analysis showed two dominant analyte peaks, which we identified as MeOH and EtOH (Fig. 1) based on the results obtained with the standard solutions.

The quantitative evaluation was carried out using PrOH as IS. The FID sensitivity towards PrOH was proven to be 1.32 and 1.43 times lower than towards MeOH and EtOH, respectively. Therefore the respective areas must be corrected after getting them using the data integration software. The calculation of the concentration of each individual compound, in mg kg^−1^, was performed as follows:

$$\text{Volatile}\;x=\frac{Ax\times mIS}{AIS\times mS}$$

where *Ax* is the peak area for the volatile *x* divided by the its correction factor, *AIS* is the area of the PrOH peak, *mIS* is the mass of PrOH added (in mg), and *mS* is the mass of the sample used for the determination (in g).

Table 2 shows the results of the analysis. Around 78% of the results show values for the relative standard deviation (referred to three times the standard deviation of the repeatability) not higher than 13%, which can be considered as quite good.

## Additional information

This procedure is a modification of the Spanish specification UNE-EN 14110 [3], to determine MeOH content in fatty acid methyl esters (FAME) utilized as biodiesel. According to this method the samples must be heated in a sealed vial at 80 °C until the equilibrium is reached. The vial headspace is then sampled and analysed by capillary GC-FID. Quantitation is carried out with a three-point calibration curve using standard FAME solutions. The procedure only quantitates the MeOH content, and the use of external standardization may represent an error source.

A method for determining MeOH and EtOH in olive oil samples by GC-FID using packed columns has been developed by Mariani and co-workers [4]. They use a modified liner in a way that they can inject the oil samples directly into the gas chromatograph. The oil is then heated and the adapted liner permits the concentration of the headspace from which the volatile fraction goes directly to the column, whereas the triglycerides remain in the liner's reservoir. Thanks to the column characteristics also in this case just MeOH and EtOH peaks are present in the chromatograms, but again the use of external standards for the quantitative determinations introduces an error source. Additional disadvantages of this method are the fact of needing an altered injector and the utilization of packed columns, which may not be so common nowadays. In any case Mariani's results on the content of MeOH (3–10 mg kg^−1^) and EtOH (1–28 mg kg^−1^) in virgin olive oils are comparable –within our error limits– to those obtained when applying the present method (Table 2), which supports the possibility of using both procedures according to the laboratory equipment.

## Figures and Tables

**Table 1:** Relative retention time (Rt) values of methanol (MeOH) and ethanol (EtOH) with respect to 1-propanol (PrOH), with three times their SD. The corresponding Rt windows (Rt ± 3SD) are also given.

	MeOH	EtOH	1-PrOH
Relative Rt	0.677 ± 0.002	0.744 ± 0.002	–
Rt window [min]	4.417 ± 0.026	4.850 ± 0.026	6.522 ± 0.034

**Table 2:** Methanol (MeOH) and ethanol (EtOH) contents in mg kg^−1^ olive oil from different cultivars. The values correspond to a minimum of two independent measures. Three times the standard deviation of the repeatability (SRr), together with the corresponding relative values (RSDr), are also given.

Olive cultivar	MeOH [mg kg^−1^]	3SDr	RSDr [%]	EtOH [mg kg^−1^]	3SDr	RSDr [%]
Adramitini	1.95	0.07	4	0.74	0.12	16
Blanqueta	2.81	0.21	7	1.16	0.09	8
Bouteillan	3.97	0.52	13	0.97	0.05	5
Chemdal Kabylie	9.43	0.93	10	1.23	0.05	4
Cipresino	3.72	0.65	18	1.07	0.06	5
Coratina	8.91	1.78	20	0.76	0.05	6
Frantoio	1.30	0.04	3	1.02	0.03	2
Koroneiki	2.22	0.28	13	0.64	0.12	19
Leccino	1.96	0.48	24	1.49	0.06	4
Lechín de Granada	5.98	0.12	2	2.07	0.10	5
Manzanilla	2.48	0.15	6	0.86	0.09	11
Negral	1.40	0.24	17	2.07	0.08	4
Pendolino	1.21	0.05	4	1.11	0.14	13
Picual	5.14	0.25	5	1.71	0.02	1
Rapasayo	2.09	0.20	9	1.79	0.32	18
Sigoise	8.25	0.80	10	1.09	0.03	3
